# Luxatio Erecta with Complex Medical Decision-Making Regarding Reduction: A Case Report

**DOI:** 10.5811/cpcem.64488

**Published:** 2026-09-08

**Authors:** Mitch Daniel, Aaron B. Deutsch, Marna R. Greenberg

**Affiliations:** * University of South Florida Health Morsani College of Medicine, Lehigh Valley Campus, Bethlehem, Pennsylvania; † Jefferson Health Lehigh Valley Region, Department of Emergency and Hospital Medicine, Division of Medical Toxicology, Bethlehem, Pennsylvania; ‡ Jefferson Health Lehigh Valley Region, Department of Emergency and Hospital Medicine, Bethlehem, Pennsylvania

**Keywords:** inferior shoulder dislocation, luxatio erecta, procedural sedation, risk-benefit, case report

## Abstract

**Introduction:**

Luxatio erecta is a rare glenohumeral dislocation. The standard treatment is reduction under procedural sedation; however, decision-making becomes more nuanced in patients with comorbidities. This report describes a hyperglycemic patient with type 1 diabetes with luxatio erecta.

**Case Report:**

An 18-year-old man resented with a traumatic inferior shoulder dislocation. Laboratory tests indicated severe hyperglycemia without diabetic ketoacidosis. The emergency department team performed a risk-benefit assessment before proceeding with procedural sedation. Closed reduction was successful, and hyperglycemia was managed with intravenous fluids.

**Conclusion:**

Inferior shoulder dislocations are rare; severe hyperglycemia in diabetics is not an absolute contraindication to procedural sedation, but the patient may need individualized risk-benefit assessment.

## INTRODUCTION

Shoulder dislocations are a relatively common orthopedic injury in which the humeral head is forced out of the glenoid socket. They are the most common form of joint dislocation due to the extensive range of motion in multiple planes allowed by the glenohumeral joint allows, with an incidence of 51.2 per 100,000 persons per year.^1^ Risk factors for shoulder dislocations are largely trauma-related, especially from contact sports like football and basketball, in which the shoulder is subject to vulnerable positions for dislocation.^2^ Treatment for shoulder dislocation comprises prompt closed reduction, paying careful attention to axillary nerve and axillary artery damage, which may present as loss of deltoid strength and lateral shoulder sensation or weak distal pulses, respectively.^3^

Most shoulder dislocations are anterior, occurring after forced external rotation and abduction of the arm and forcing the humeral head to displace anteriorly out of the “socket,”^2^,^4^ This often occurs during contact sports or trauma.^2^ However, other forms of shoulder dislocation may occur depending on patient anatomy, mechanism of injury, and comorbidities. For example, epileptic patients are prone to posterior shoulder dislocations because powerful, involuntary contractions of internal rotator and adductor muscles overpower the same involuntary contractions of opposing muscles, forcing the humeral head to displace posteriorly against the glenoid rim.^5^,^6^

A small subset of patients present with inferior shoulder dislocations, also known as luxatio erecta. This condition refers specifically to an inferior dislocation of the glenohumeral joint in which the humeral head becomes trapped beneath the coracoid process and glenoid rim. It is a very rare subtype, accounting for approximately 0.5% of all shoulder dislocations.^7^ One proposed mechanism of action is forceful hyperabduction causing the humeral head to lever down.^8^ Another proposed mechanism of action is direct axial force on an abducted arm.^8^

Inferior shoulder dislocations are typically treated in the same manner as other shoulder dislocations: closed reduction followed by shoulder immobilization and pain management.^2^,^9^ Conscious sedation may involve a combination of benzodiazepines, opioids, and nerve blocks, making management patient-dependent. Many situations exist in which the full medical combination is relatively contraindicated or not recommended, and these decisions are largely based on American Society of Anesthesiologist (ASA) guidelines.^10^ For example, patients with significant cardiorespiratory comorbidities undergoing procedural sedation face an increased risk as high as 14.7% for adverse respiratory events, versus 0% with peripheral nerve block alone (*P* < .001).^10^ Another relative contraindication is urgent situations in patients at risk for pulmonary aspiration because there is no opportunity for fasting.^11^

The 2018 ASA Practice Guidelines for Moderate Procedural Sedation and Analgesia consider an ASA physical status of 1 or 2 to be appropriate for procedural sedation.^11^ An ASA physical status of 1 or 2 refers to a healthy patient with no comorbidities or a patient with mild, well-controlled disease, respectively. Patients with an ASA physical status of 4 (severely compromised or medically unstable) are generally not candidates for procedural sedation, and consultation with an anesthesiologist is recommended. However, ASA status 3 (moderate to severe systemic disease) is a gray area in that procedural sedation is not a hard contraindication, but a careful risk-benefit assessment is necessary beforehand. Examples of a patient who is classified as ASA 3 would be an individual with coronary artery disease with a previous myocardial infarction, or it could be as simple as morbid obesity (Figure).

If a patient is healthy or has only mild disease, procedural sedation in the emergency department (ED) prior to reduction is appropriate. Alternatively, if the patient has severe, systemic disease that is unstable, an anesthesia consultation should be sought.

According to the American Diabetes Association (ADA), procedural sedation in patients with hyperglycemia is reasonable following adequate preprocedural assessment including screening for hyperglycemic emergency. If there are no concerns for hyperglycemic emergency and no underlying organ failure, normalizing periprocedural blood glucose is the main goal.^12^ Data on sedation agent selection for hyperglycemic patients are limited, but universal principles of avoiding respiratory depression and maintaining hemodynamic stability apply. Therefore, the choice between procedural sedation and an anesthesia consultation should be individualized. This report highlights a young man with type 1 diabetes (T1D) who presented to the ED with an inferior shoulder dislocation following a motor vehicle collision (MVC). On admission the patient’s glucose level was uncontrolled. We present this case as a borderline ASA III example in which the decision to proceed with procedural sedation was made and proved successful.

*CPC-EM Capsule*

What do we already know about this clinical entity?

*Luxatio Erecta is a rare glenohumeral dislocation. The standard treatment is reduction under procedural sedation, however, decision making becomes more nuanced in patients with comorbidities.*

What makes this presentation of disease reportable?

*An 18-year-old male presented with a traumatic inferior shoulder dislocation. His labs indicated severe hyperglycemia. Risks versus benefits in the approach to reduction are discussed.*

What is the major learning point?

*Severe hyperglycemia in diabetics is not an absolute contraindication to procedural sedation, but the patient may need individualized risk-benefit assessment.*

How might this improve emergency medicine practice?

*Physicians benefit from familiarity with the American Society of Anesthesiologist risk categorizations. This case reviews the guidelines of approach for patients requiring sedation.*

## CASE REPORT

An 18-year-old man with a history of T1D presented with right shoulder injury following a moderate-speed MVC. He was an unrestrained passenger in a truck that swerved off the road. The patient was able to self-extricate, and there was no loss of consciousness. He presented with severe right shoulder pain and a clearly visible deformity. The patient was placed in a cervical collar, and there were no signs of hemodynamic instability on primary survey.

On secondary survey, there was obvious tenderness to the right shoulder and upper arm. The patient’s right arm was held in a position above his head, mimicking a raised hand, and he was unable to voluntarily lower his arm. Grip strength of the upper right extremity was 5/5. Laboratory tests indicated a glucose level of 496 mg/dL (reference range, 70–99 mg/dL), and the patient’s mother reported that he was noncompliant with his insulin regimen at home, causing his sugars to run high consistently. Treatment options for hyperglycemia, including intravenous (IV) fluids and insulin administration, were discussed with the patient. He preferred to initiate management with IV fluids and glucose monitoring, noting that he had not eaten for at least six hours. From a clinical standpoint, this approach was considered reasonable, particularly in the setting of anticipated procedural sedation, where avoiding iatrogenic hypoglycemia was a priority. Laboratory tests were otherwise unremarkable, and there were no concerning physical signs of diabetic ketoacidosis. On exam, the patient had no abdominal pain, fruity odor on his breath, rapid/deep or labored breathing (Kussmaul respirations), confusion, or decreased alertness. Two IV lines were placed, and he received IV fluids and 75 mcg of fentanyl for significant pain and discomfort. Chest and right shoulder radiographs revealed a right shoulder inferior dislocation (luxatio erecta) with no evidence of fractures (Image 1).

Considering a regional block approach to be safer in context of the scenario, an intraarticular injection of 1% lidocaine (without epinephrine) in the right shoulder was performed. The attempt at reduction after the regional block was unsuccessful, and procedural sedation was initiated with ketamine (60 mg) and midazolam (1 mg). The patient’s right shoulder was subsequently reduced using traction-countertraction. The procedure was performed without difficulty or complication. Postprocedure, his right radial pulse was 2+ with a brisk capillary refill; he was able to give a thumbs up and he voiced normal sensation of his right hand. These physical examination findings helped support the presence of intact neurovascular structures. Postreduction computed tomography of the right shoulder suggested successful reduction of the luxatio erecta, but a comminuted greater tuberosity fracture was also discovered (Image 2). The patient was placed in a shoulder immobilizer postreduction.

Two and a half hours postreduction, the patient’s glucose level was 340 mg/dL (reference range, 70–99 mg/dL). He was educated on the importance of glucose control, and after consulting orthopedics, he was discharged with an outpatient orthopedic follow-up scheduled.

## DISCUSSION

Inferior shoulder dislocation, or *luxatio erecta*, is a rare glenohumeral dislocation, and forceful hyperabduction is one mechanism in which this injury can occur.^7^ However, for our patient, direct axial load on an abducted arm that was bracing for impact in the setting of a MVC was likely the case. One interesting point of discussion surrounding inferior shoulder dislocations involves associated secondary injuries. Although understudied, a retrospective case series of 38 cases of luxatio erecta suggests an increased risk of concomitant injuries, potentially even greater than that seen in anterior shoulder dislocations.^14^ Associated injuries can include Hill-Sachs lesions, greater tuberosity fractures, rotator cuff injuries, and neurovascular damage. The case series reports associated pathologies in 81% of inferior shoulder dislocations versus 60% of anterior shoulder dislocations and, more specifically, the authors reported an increased rate of associated greater tuberosity fractures in 34% of the patients.^14^

The decision for closed reduction under conscious sedation, which is the mainstay of treatment for shoulder dislocations, was a success. Although conscious sedation is frequently employed, we recommend an individualized approach when foregoing anesthesia consultation in patients who are borderline ASA physical status 3 or greater. This patient’s severe hyperglycemia in the setting of T1D raised concern for periprocedural risk. According to ADA and ASA guidelines, hyperglycemia does not contraindicate procedural sedation, provided that hyperglycemic emergencies have been ruled out and the patient is stable. This approach is supported by the American College of Emergency Physicians (ACEP) policy statement on unscheduled procedural sedation guidelines, which uses the ASA risk assessment as a cornerstone of its recommendations.^16^ Clearly patients with severe systemic disease (greater than ASA physical status 3) are at greater risk for adverse events.^17^–^19^ The ACEP guidelines endorse that even in high-risk patients, circumstances remain in which time-sensitive sedation is necessary. 16 This approach is particularly appropriate when resources do not permit the timely availability of an anesthesiologist or operating room without the risk of harm to the patient.^16^ Additionally, this patient’s otherwise normal laboratory values (specifically, the absence of acidosis), young age, and lack of comorbidities supported the decision for sedation in the ED. Ultimately, it was decided that the urgency of treating the dislocation outweighed the case-specific risks associated with conscious sedation for our patient.

Deferring insulin administration for the patient’s initial hyperglycemia was clinically appropriate given the anticipated course of procedural sedation and his fasting state of more than 6 hours. Insulin could have been given to treat the hyperglycemia; however, this intervention would have increased the risk of iatrogenic hypoglycemia. Hypoglycemia-related neurological changes could be difficult to distinguish from typical sedation effects. In addition, a postprocedural neurological assessment of the right upper extremity could also be affected by hypoglycemia. Initial management with IV fluids alone can lead to significant glucose reduction by improving renal glucose clearance through increased renal perfusion and volume expansion,^15^ as was observed in this patient. His glucose began trending down postreduction after the procedure. This self-resolution with IV fluids and the absence of other laboratory or physical examination abnormalities suggest a few possibilities, the most likely of which is that the patient had isolated hyperglycemia in the setting of insulin nonadherence or stress-induced hyperglycemia.The clinical team appropriately assessed and treated the patient’s hyperglycemia in this situation.

From a procedural standpoint, intraarticular lidocaine followed by ketamine and midazolam were appropriate medication choices that aided in a successful reduction without complications. Midazolam was administered to reduce the risk of ketamine-associated emergence reactions. Regarding sedation choices and their impact on hyperglycemia, the published reports conflict. Several studies have shown that patients receiving midazolam and propofol experience increased blood glucose levels; however, at least one study evaluating blood glucose responses in light procedural sedation cataract surgery using either agent with fentanyl found no difference in blood glucose alterations.^20^–^23^ There is consensus across these studies that avoidance of hypoglycemia is crucial, and that procedural stress, pain, and local anesthetics with epinephrine can increase blood glucose levels.^20^–^23^ Although data guiding sedative agent selection in patients with significant hyperglycemia are limited, this case supports the safe use of ketamine with benzodiazepine adjunct, because no adverse events occurred.

## CONCLUSION

While rare, luxatio erecta carries the risk of associated fractures and neurovascular injury that may not be initially apparent on imaging. Additionally, this case illustrates that while an ASA physical status of 3 is not an absolute contraindication to conscious sedation, it does necessitate careful assessment of patient stability and sedation strategies. This case demonstrates that closed reduction with conscious sedation can be safely performed in a young, otherwise healthy, severely hyperglycemic T1D patient with an inferior shoulder dislocation. However, individualized sedation decision-making in patients with an ASA physical status of 3 in the ED is recommended.

## Figures and Tables

**Figure.**
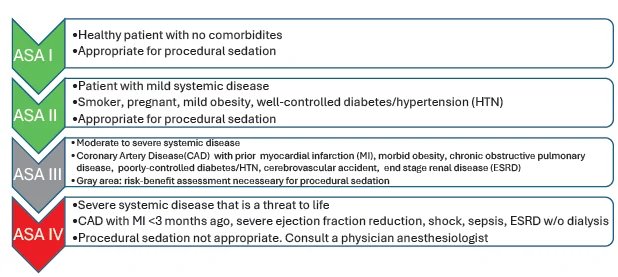
American Society of Anesthesiologist risk classifications (Practice Guidelines 2018). *ASA*, American Society of Anesthesiologists

**Image 1:**
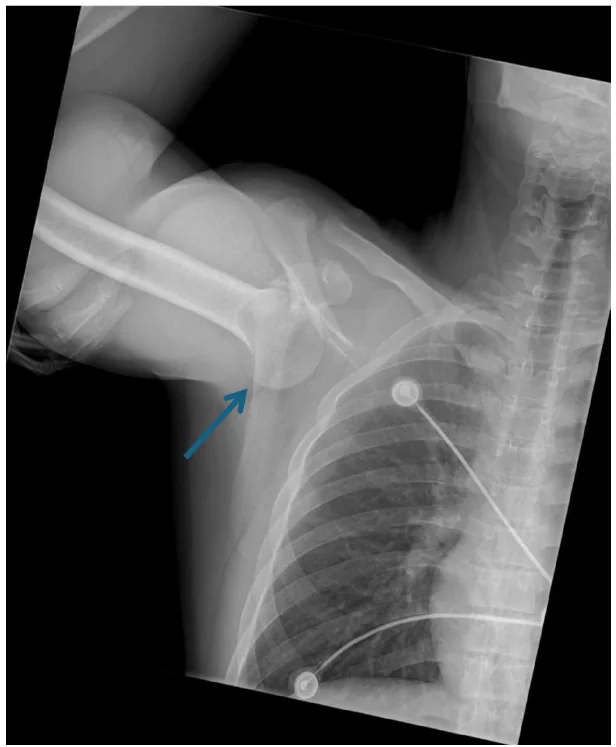
Right shoulder radiograph showing inferior shoulder dislocation.

**Image 2:**
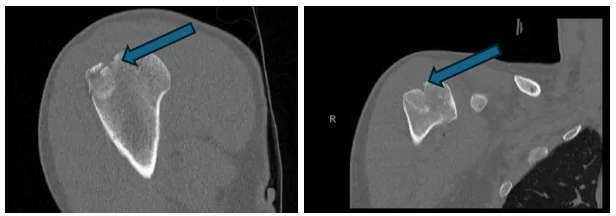
Right shoulder computed tomography showing comminuted greater tuberosity fracture.
